# Establishment of a High-throughput Setup for Screening Small Molecules That Modulate c-di-GMP Signaling in *Pseudomonas aeruginosa*

**DOI:** 10.3791/54115

**Published:** 2016-06-30

**Authors:** Kushal N. Rugjee, Shi-qi An, Robert P. Ryan

**Affiliations:** ^1^Division of Molecular Microbiology, College of Life Sciences, University of Dundee

**Keywords:** Immunology, Issue 112, Cyclic di-GMP signaling, high-throughput screen, green fluorescent protein reporter, antibiotic resistance, biofilm formation, *Pseudomonas aeruginosa*

## Abstract

Bacterial resistance to traditional antibiotics has driven research attempts to identify new drug targets in recently discovered regulatory pathways. Regulatory systems that utilize intracellular cyclic di-GMP (c-di-GMP) as a second messenger are one such class of target. c-di-GMP is a signaling molecule found in almost all bacteria that acts to regulate an extensive range of processes including antibiotic resistance, biofilm formation and virulence. The understanding of how c-di-GMP signaling controls aspects of antibiotic resistant biofilm development has suggested approaches whereby alteration of the cellular concentrations of the nucleotide or disruption of these signaling pathways may lead to reduced biofilm formation or increased susceptibility of the biofilms to antibiotics. We describe a simple high-throughput bioreporter protocol, based on green fluorescent protein (GFP), whose expression is under the control of the c-di-GMP responsive promoter *cdrA*, to rapidly screen for small molecules with the potential to modulate c-di-GMP cellular levels in *Pseudomonas aeruginosa *(*P. aeruginosa*). This simple protocol can screen upwards of 3,500 compounds within 48 hours and has the ability to be adapted to multiple microorganisms.

**Figure:**
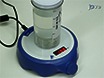


## Introduction

The rapid development of bacterial resistance against clinically important antibiotics is one of the major concerns currently facing health professionals worldwide. This failure of traditional antibiotics has driven new searches for chemical matter that can interfere with bacterial processes involved in virulence and disease progression^1^. One such regulatory system that utilizes the intracellular second messenger cyclic di-GMP (c-di-GMP) has recently become a target with promising validity^2-4^. It has been established that this global second messenger signal molecule regulates many functions including antibiotic resistance, adhesion, biofilm formation and disease^2-4^.

It is now understood that the cellular level of c-di-GMP in the bacterial cell is controlled by synthesis and degradation whereby two molecules of GTP are used to synthesize c-di-GMP by GGDEF domain-containing diguanylate cyclases (DGCs) whereas c-di-GMP degradation is catalyzed by phosphodiesterases (PDEs) that have either an EAL or an HD-GYP domain (reviewed in (3, 5)). Proteins containing these domains often contain other signaling domains, suggesting that their activity in c-di-GMP turnover is regulated either directly or indirectly by environmental or cellular cues^3,5^. Consequently, c-di-GMP signaling functions to link the sensing of diverse environmental cues to modifications in bacterial phenotype. c-di-GMP exerts its regulatory effect in bacteria at the level of transcription, post-transcription and post-translation by various mechanisms^4^.

A major influence of c-di-GMP in many bacterial cells is in the determination of bacterial 'lifestyle' and, in particular, in the control of transitions between motile planktonic cells and sessile cells attached to surfaces or organized in the multicellular structures of biofilms^3,5^. In general, high cellular levels of c-di-GMP are associated with biofilm formation and sessility, while low cellular levels encourage motility and virulence factor synthesis in many bacterial pathogens^3,5^. Thus, a more detailed knowledge of the workings of c-di-GMP signaling could afford strategies for inhibition of biofilm formation and virulence in bacterial pathogens. This is a daunting task given that most bacterial genomes encode numerous proteins with GGDEF, EAL and/or HD-GYP domains (for example *P. aeruginosa* has over 40 proteins) and multiple effectors^6,7^.

However, even with this complexity, recent evidence suggests that strategies manipulating c-di-GMP signaling may be developed to either prevent antibiotic resistant infections developing or make them susceptible to the immune system or efficient treatment by co-administration of classic antibiotics^2^. In line with this, it has been experimentally demonstrated that artificial decrease of intracellular c-di-GMP in *in vitro*-grown *P. aeruginosa* leads to decreased biofilm formation and increased susceptibility to antimicrobials, while *P. aeruginosa*-developed biofilms on silicone implants, located in the peritoneal cavity of mice, can be dispersed in a similar fashion^8-11^.

Here, we describe a high-throughput, fluorescence-based reporter assay to screen small molecules that can potentially modulate the cellular c-di-GMP levels in *P. aeruginosa *(**Figure 1**). The assay is based on measuring c-di-GMP cellular levels using a previously developed GFP reporter whose expression is transcriptionally linked to the c-di-GMP-responsive *cdrA* promoter^12^. This protocol describes the methodology for expression of the reporter construct in the *P. aeruginosa* strain of interest, compound plate preparation, culture inoculation into 384-well plates, growth conditions, as well as details regarding data collection, management and analysis (**Figure 1**). Overall, this protocol will aid researchers to potentially identify novel compounds targeting c-di-GMP signaling in bacteria, and for use in research aiming at understanding the biology of *P. aeruginosa*.

## Protocol

Note: Procedures for cloning and transformation of the purified c-di-GMP reporter plasmid are discussed elsewhere^12^.

### 1. Generation of GFP Tagged c-di-GMP Reporter *P. aeruginosa *Strain

1. Inoculate 5 ml of lysogeny broth (LB) medium with a single colony of *P. aeruginosa* and incubate overnight at 37 °C with shaking at 200 rpm.
2. Transfer 2 ml of the overnight culture into 200 ml of fresh LB medium in a 1 L flask and incubate at 37 °C with shaking at 200 rpm.
3. Monitor optical density at 600 nm (OD_600_) every 30 min by taking a 1 ml sample from the flask and examine using a spectrophotometer (as described previously^12^).
4. When the OD_600_ reaches between 0.3 - 0.5, place 40 ml of the culture into a 50 ml conical centrifuge tube.
5. Pellet the cells by centrifuging at 8,000 rpm for 10 min at 4 °C, discard the supernatant and re-suspend the cells in 40 ml of ice-cold, sterile, 300 mM sucrose solution.
6. Pellet the cells a second time by centrifuging as described in step 1.5 and re-suspend in 20 ml of ice-cold, sterile, 300 mM sucrose solution.
7. Pellet the cells a final time by centrifuging as described in step 1.5 and re-suspend in 400 µl of ice-cold, sterile, 300 mM sucrose solution. Note: The cell density at this point should be approximately 1 x 10^11^ colony forming units per milliliter (CFU/ml).
8. Chill the cells on ice for 30 min, after which they are ready for electroporation.
9. Add 1 µl of a 0.2 µg/µl pCdrA::*gfp*^C^ plasmid ^12 ^solution to 40 µl of *P. aeruginosa* electro-competent cells prepared above in a 1.5 ml microcentrifuge tube that has been pre-chilled on ice. Mix the suspension and transfer into a pre-chilled, 2 mm electrode gap, electroporation cuvette. Note: pCdrA::*gfp*^C^: pUCP22Not-P*_cdrA_*-RBSII-*gfp*(Mut3)-T_0_-T_1_, Amp^r^ Gm^r^, which gives a fluorescent readout of the intracellular level of c-di-GMP in *P. aeruginosa *strains, was developed and validated by Rybtke *et al.*^12^.
10. Remove moisture on the outside of the cuvette with tissue paper and place the cuvette into the sample chamber of the electroporator. Pulse with a voltage of 2.5 kV, capacitance of 25 µF and resistance of 200 Ω.
11. Remove the cuvette and add 1 ml of LB medium. Then, transfer the cells to a sterile 1.5 ml microcentrifuge tube and incubate for 2 hr at 37 °C with shaking at 200 rpm.
12. Spread 10 µl, 50 µl and 100 µl aliquots of the culture onto sterile LB-agar plates supplemented with ampicillin (at a concentration of 100 µg/ml), and incubate the plates at 37 °C overnight.
13. Confirm the GFP expression (excitation at 485 nm, emission at 520 nm) by examining the plate under a fluorescence microscope with a standard GFP channel and pick a single colony to inoculate 5 ml LB. Incubate overnight as described in step 1.1. Mix 0.5 ml of the overnight culture with 0.5 ml 50% glycerol in a 2 ml screw top tube and store at -80 °C.

### 2. Preparation of the Starter Culture for Inoculation

1. Two days before the screen, plate the *P. aeruginosa* strain (containing the pCdrA::*gfp*^C^ plasmid) from the -80 °C stock onto an LB-agar plate (supplemented with ampicillin at a concentration of 100 µg/ml) by gently spreading the bacteria over the plate using a sterile inoculation loop. Incubate the plate overnight at 37 °C.
2. The evening before the screening, inoculate a single colony of *P. aeruginosa* from the plate into 10 ml of LB medium (supplemented with ampicillin at a concentration of 100 µg/ml) in a tube and incubate the pre-culture overnight at 37 °C with shaking at 200 rpm.
3. On the day of the screen, prepare a subculture from the overnight pre-culture by diluting it first with fresh LB medium to an OD_600_ of 1.0 and then further diluting with 5% LB to an OD_600_ of 0.04 (1:25 ratio). Note: The total volume of the inoculated medium depends on the number of plates to be tested. 5% LB is made by diluting 100% LB with phosphate buffered saline (PBS) to the required concentration. Importantly, the media (5% LB) allows constitutive activation of pCdrA::*gfp*^C^ in *P. aeruginosa*.
4. Add a sterile magnetic stirrer bar into the container and stir the culture at minimal speed on a magnetic stirrer for 30 min at room temperature, allowing the bacteria to acclimatize to the media before they are dispensed into 384-well plates.

### 3. Inoculation and Incubation of the 384-well Plates Containing Small Molecules

Note: Sterility and good aseptic techniques are paramount to the following steps.

1. To prepare the positive control (100% inhibition), add 20 µl tobramycin sulfate (25 mg/ml) to 10 ml (adequate for up to 12 plates) of the subculture prepared in Step 2.3 and mix gently. Pipette 40 µl of the positive control culture into wells A23 - P23 (**Figure 2**).
2. To prepare the negative control (0% inhibition), add 30 µl dimethyl sulfoxide (DMSO) to 10 ml (adequate for up to 12 plates) of the subculture prepared in Step 2.3 and mix gently. Pipette 40 µl of the negative control culture into wells A24 - P24 (**Figure 2**).
3. For each of the plates stamped with the small molecules, inoculate a volume of 40 µl of the diluted overnight cultures (described in Step 2.3) into the wells A1 - P22 (**Figure 2**). Note: This can be achieved using a liquid handler robot. Due to the heterogeneous properties of bacterial cultures, it is important to maintain a continuous and moderate agitation to keep bacteria consistently distributed in the media while dispensing. To do this, keep stirring the acclimatized culture from step 2.4 magnetically during dispensing.
4. Seal the plates with an air-permeable cover seal and incubate for 6 hr at 37 °C. Note: The air-permeable seal is critical to maintain unchanging conditions across all 384 wells and to circumvent the potential development of oxygen gradients across the plate.

### 4. Measurement of Growth (OD_600_) and Intracellular c-di-GMP Level (GFP)

1. Approximately 30 minutes before spectrophotometric measurement, pre-warm the plate reader to 37 °C to avoid condensation.
2. Gently remove the air-permeable cover seal before reading. Measure the optical density at a wavelength of 600 nm with a setting of 10 flashes per well and a settling time of 0.2 sec.
3. Prior to measuring fluorescence, make an automatic gain and focal adjustment based on well A24 (negative control) and set the gain target value at 75% (**Figure 2**).
  1. From the plate reader control software, click on start measurement and click on the ''Focus and Gain Adjustment/Plate IDs'' tab.
  2. Select ''Focus Adjustment'' and ''Channel A'' on the right on the window.
  3. Select ''Gain Adjustment'' and ''Selected well'', and type in ''75'' as the ''Target value''. Finally, pick well A24 in the plate layout before clicking on ''Start Adjustment''. Once the adjustment is carried out, click on ''Start measurement''.
  4. Measure the fluorescence from the GFP reporter at an excitation/emission maxima of 485/520 nm with a setting of 10 flashes per well and a settling time of 0.2 sec.
4. Collect data from all plates examined. Note: Data readings are automatically saved as default by the plate reader control software.
  1. View readings by clicking on the "Mars" icon within the control software to open the statistical analysis package.
  2. Retrieve data by "double clicking" on the plate number of interest to access the data for analysis.

### 5. Data Analysis

1. Evaluate the uniformity and reproducibility of the assay using robust z' analysis, which is the most common quality metrics reported for high-throughput screens. Calculate robust z' using the formula: 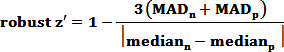 Where MAD (Median Absolute Deviation) is an estimate of standard deviation, p is the positive control (100% inhibition) and n is the negative control (0% inhibition) for both OD_600_ and GFP values. Note: An assay with a robust z' value (calculated for both OD_600_ and GFP raw data) greater than or equal to 0.5 is considered as an excellent assay.
2. Calculate% inhibition of growth using the formula: 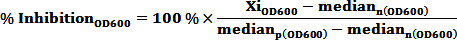 Where Xi_OD600 _is the iterated OD_600 _value of each sample well. % Inhibition_OD600 _> 0, growth inhibitor; % Inhibition_OD600 _< 0, growth promoter.
3. Assess the % inhibition of the intracellular level of c-di-GMP using the formula (the change in arbitrary fluorescence intensity units are corrected for the change in cell density): 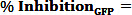 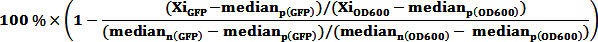 Where Xi_GFP_ is the iterated GFP value of each sample well. % Inhibition_GFP _> 0, c-di-GMP inhibitor; % Inhibition_GFP _< 0, c-di-GMP promoter.
4. Set a threshold for hit detection at 3 MAD from the median (median ±3 MAD), calculated from the sample well read-outs of each plate, assuming a normal distribution of data-points. Note: However, a ±50% inhibition cut-off can be selected for more reproducible and accurate dose response assays if required.

## Representative Results

The approach described here allows a single researcher to efficiently and successfully screen an average of 3,500 compounds within a 48-hour period. An overview of the high-throughput screening protocol is illustrated as a flowchart in **Figure 1**. For the current article, we screened a rule-of-five compliant compound library for potential c-di-GMP modulating compounds at a final concentration of 600 µM. However, there are many commercially available drug-like and non-drug like compound sets that could be used in the assay. These sets can be bacteria-focused compounds or random pooled compounds but each library would have predicated physicochemical properties and characteristics assigned such as the set screened here, which had polar functional groups and multiple stereogenic centers. In this protocol, bacteria are inoculated into the plate along with compounds, an antibiotic positive control and a DMSO vehicle control, according to the layout shown in **Figure 2**. **Figure 3** depicts the results of the primary screening exemplified by a single library plate. Representative OD_600 _and GFP raw data are shown in **Figure 3A** and **3B**, respectively. A color gradient heat map, with hot (red) to cool (green) colors indicating low to high OD_600_/GFP values, has been applied to the well values. The heat maps were generated in a spreadsheet software by applying a 3-Color scale conditional formatting spanning from the lowest OD_600_/GFP read-out up to the highest OD_600_/GFP read-out. Low OD_600_ and GFP values relative to the DMSO negative control correspond to an inhibition in growth and c-di-GMP levels respectively, while high values relative to the DMSO negative control correspond to a promotion in growth and c-di-GMP levels respectively. The robust z' values for OD_600_ and GFP are calculated according to the formula provided in the protocol (5.1). As they are both above 0.5 (0.694 and 0.761 respectively), the assay quality was deemed robust and the data was further analyzed. The % inhibition of growth (OD_600_) and intracellular c-di-GMP level (GFP) are calculated by the formulae provided in the protocol (5.2, 5.3). Representative data are shown in **Figure 4A** and **4B** respectively. A color gradient heat map, with hot (red) to cool (green) colors indicating low to high % inhibition, has been applied to the well values. A scatter plot of the % inhibition_(OD600/GFP) _from individual wells based on the primary screen is plotted in **Figure 5A** and **5B** for OD_600_ and GFP data respectively. A ±50% cut-off (indicated with dotted lines) is selected for hit detection. Potential hits based on this cut-off are indicated with red dots. Two types of compounds of interest can be discerned from the assay. Hits identified in **Figure 5A** are compounds that inhibit bacterial growth, while hits identified in **Figure 5B** are compounds that potentially possess the ability to modulate intracellular levels of c-di-GMP. Two compounds have been identified as representative hits for this assay. These are compound A, a potential growth inhibitor and compound B, a potential c-di-GMP inhibitor. Compound A corresponds to well F8 in **Figures 3 **and** 4** and had a 72.5% inhibition in growth. There was an expected corresponding inhibition in cyclic di-GMP levels. Compound B corresponds to well K3 in **Figures 3 **and** 4** and had a 61% inhibition in cyclic di-GMP levels with no change in growth. Select hit compounds were further tested by a 10-point dose-response assay with a top concentration of 2 mM and two-fold dilution series. IC_50_ values are calculated based on the dose-response curves (**Figure 6A and B**).


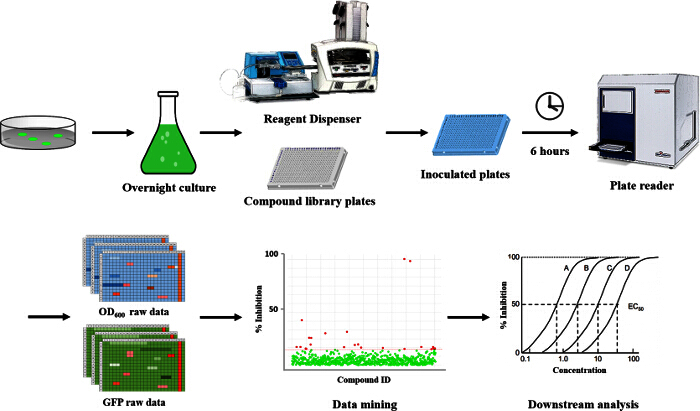
**Figure 1.Overview: High-throughput Setup for Screening Small Molecules for Their Potential to Modulate Cellular Levels of c-di-GMP in*****P. aeruginosa*****.** This overview shows a step-by-step representation of the protocol used in this assay. A bacterial colony of *P. aeruginosa* is grown overnight in an LB starter culture. Using a reagent dispenser, the cells are inoculated into 384-well plates containing the selected compounds. The plates are incubated for 6 hours, after which the OD_600_ and GFP values are measured. Using these read-outs, compounds that affect the cellular levels of c-di-GMP can be identified. Please click here to view a larger version of this figure.


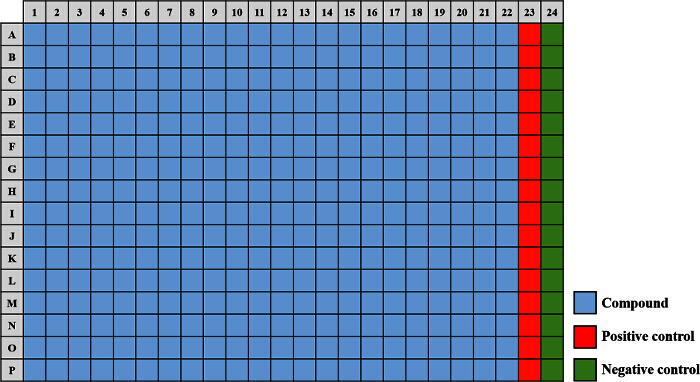
**Figure 2.A 384-well Plate Map Used for the Small Molecule Screening Assay.** The compounds from the library (light blue) are aliquoted into wells A1 - P22. The "positive control" (red) consisting of a final concentration of 50 µg/ml tobramycin sulfate is added to wells A23 - P23 and the "negative control" (green) containing a final concentration of 1% DMSO is added to wells A24 - P24. Please click here to view a larger version of this figure.


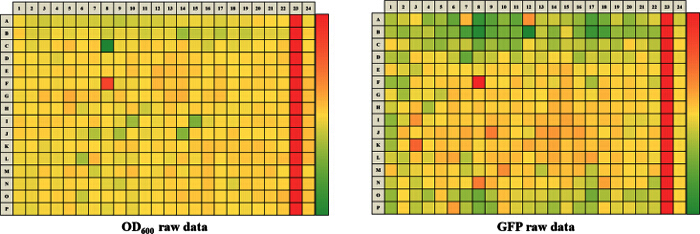
**Figure 3.Representative Results for One 384-well Screening Plate.** Representative raw data is for OD_600_ (A) and GFP (B). A color gradient heat map, with hot (Red: OD_600 _= 0.65 or GFP = 250,000) to cool (Green: OD_600 _= 0.10 or GFP = 40,000) colors indicating low to high values, has been applied to the well values. Wells that have lower OD_600_/GFP readings relative to the DMSO negative control will tend to be in red while wells that have higher OD_600_/GFP readings relative to the DMSO control will tend to be in green. Please click here to view a larger version of this figure.


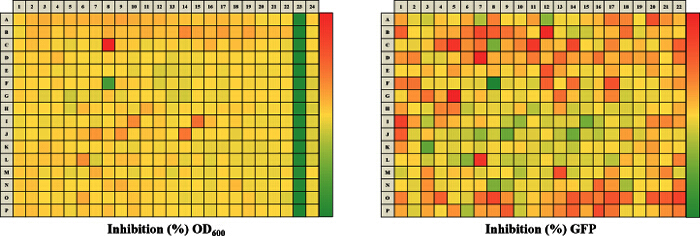
**Figure 4.Representative Results for Percentage Inhibition Calculated for a 384-well Plate.** The analyzed % inhibition data is represented for both of the OD_600_ (A) and GFP (B) read-outs. A color gradient heat map, with hot (Red: OD_600 _= -150% or GFP = -20%) to cool (Green: OD_600 _= 100% or GFP = 100%) colors indicating low to high inhibition values, has been applied to the well values. Small molecules, which potentially inhibit growth and intracellular c-di-GMP will tend to be in green while small molecules which potentially promote growth and intracellular c-di-GMP levels will tend to be in red. Please click here to view a larger version of this figure.


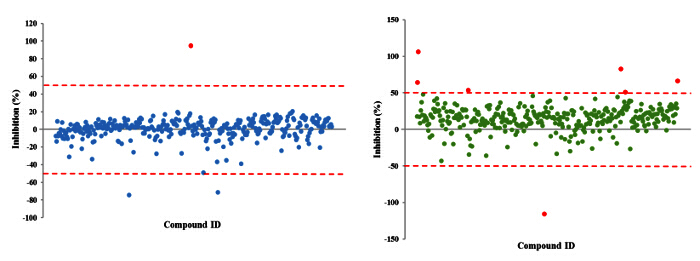
**Figure 5. Representative Scatter Plots for % Inhibition_(OD600/GFP) _Obtained From Each Tested Small Molecule. **Each small molecule is represented by a dot and the % inhibition distribution for each of the OD_600_ (A) and GFP (B) read-outs is shown. A ±50% cut-off is selected for hit identification. Potential hits are highlighted in red. Please click here to view a larger version of this figure.


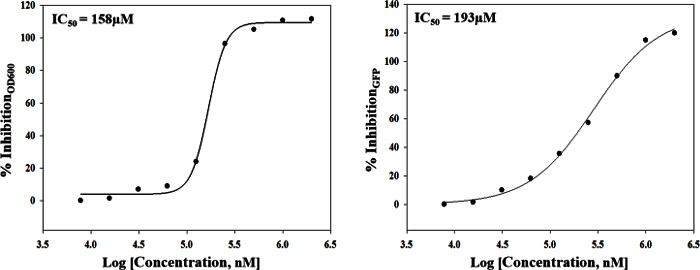
**Figure 6.Representative Results From a Dose - Response Assay.** Two compounds (potential growth inhibitor A and potential c-di-GMP inhibitor B) identified from previous screens are further tested in a 10 point dose-response assay with a top concentration of 2 mM and two-fold dilution series. Fitting a four-parameter logistic function to the data yielded IC_50_ values of 158 µM and 193 µM respectively. Please click here to view a larger version of this figure.

## Discussion

In order to improve the treatment of bacterial infections, it is clear that a better understanding of bacterial behavior at the molecular regulatory level is required. The procedure described here will be beneficial for microbiologists, biochemists and clinicians who want to uncover small molecules that have the potential to manipulate or interfere with cellular concentrations of c-di-GMP in bacteria. The method utilizes a recently developed GFP bioreporter to monitor cellular levels of c-di-GMP in *P. aeruginosa*^12^. This bioreporter has been validated and shown importantly not to affect the growth of the strain used when cultured in 5% LB in PBS. The use of a whole-cell screen to identify small molecules that alters c-di-GMP levels *in vivo* overcomes the major difficulties of target-based drug discovery in terms of molecule penetration through the bacterial membranes, particularly through those of Gram-negative bacteria. Importantly, the assay appears very robust as a robust z' value consistently above 0.5 was observed in all screens to date. Screening using this protocol will reveal a number of small molecules that inhibit and/or promote intracellular c-di-GMP levels in *P. aeruginosa*. Moreover, this assay also has the potential to identify bactericidal or bacteriostatic compounds resulting in a decrease in the OD_600_ read-out.

Although not discussed in the protocol section, there are several important considerations for the preparation of the experiment. It is vital to bear in mind that the GFP bioreporter is based on a plasmid. Although the reporter plasmid is known to be very stable in* P. aeruginosa*, it can be lost after continuous re-plating, hence the need to use freshly plated strains from a -80 °C glycerol stock, and checking fluorescence expression is critical. It is also very valuable to maintain uniform growth conditions throughout the screen since any fluctuations in these conditions could have knock-on effects on the screen. This would include making certain that media and antibiotics are premade in batch and used throughout the course of the screen. Bacterial cultures not growing (based on OD_600_ read-outs) in a uniform fashion is a common issue for most high-throughput screens of this nature. This can be due to edge effects or dispensing a non-homogeneous bacterial culture into the plates. For the former, making sure a gas-permeable seal is used during incubation is vital. While for the latter, priming the liquid handler tubing with a volume of the culture at least three times the dead volume of the tubing itself is recommended. It is crucial to keep the magnetic stirrer at minimal speed during dispensing. While monitoring and storage of the 384-well plates for consistent growth over the course of the experiments is paramount, a situation to avoid is the stacking of plates during incubation as it can cause unwanted oxygen gradients leading to a skew in the growth pattern. It is also important to ensure that the DMSO vehicle used as a negative control is not negatively impacting growth of the strain of interest. Many of these issues associated with growth can be avoided by performing a mock screen with the bacterial strain of interest prior to screening. Data interpretation also merits consideration given that c-di-GMP inhibition results are calculated by the change in arbitrary fluorescence intensity units that must be corrected for the change in cell density. With this in mind different mathematical formulas to assess the data output from these experiments should also be considered. For example, a compound could appear as a c-di-GMP inhibitor but actually be a growth inhibitor or vice versa, requiring prudent interpretation of identified hits.

There are several drawbacks and limitations that must be considered during the development and performance of this high-throughput cell-based screen. For example, the bio-reporter functions on an indirect measure of cellular c-di-GMP levels using a fluorescent probe whose detection properties could potentially be affected by small molecules used in a screen. A tangential issue is the fact that the cell-based assay gives no information regarding the mechanism behind changes in the levels of intracellular c-di-GMP. Therefore, important observations from experiments must be confirmed using a high-performance liquid chromatography-mass spectrometry approach, which is considered the "gold-standard" method for measuring intracellular c-di-GMP fluctuations in bacteria. Moreover, our procedure can only provide information regarding the behavior of bacteria grown *in vitro*, as bacterial cells grown in the context of the host (*in vivo*)**quickly modify their activities due to this environment. Furthermore, the process of using a GFP bioreporter means the screen does not take into account the physiological status of the bacterial cells. However, the protocol could be adapted to microscopic monitoring of individual wells during the course of the screen.

Even with these considerations and limitations, this high-throughput assay is still a robust screen for small molecules capable of interfering with intracellular c-di-GMP levels. Since many microbial species including many bacterial pathogens have been studied in order to characterize the modulation of intracellular c-di-GMP levels, our protocol could be applied to diverse bacterial species and expanded to the study of more complex multispecies bacteria models. The assay can be easily optimized for other bacterial species by changing the growth conditions used, or can be adapted to read other outputs by using different bioreporters. Different incubation times can be applied depending on the growth phase of interest. However, when scaling up or increasing through-put, it is important to keep in mind that the bacteria will still be growing during plate preparations and read-out measurements. Therefore it is recommended to screen a maximum of 15 plates at a time using this protocol. Moreover, using plates with more than 384 wells may not allow uniform growth, requiring further optimization. When scaling down the number of plates, it may be more appropriate to manually inoculate using an electronic pipette instead of a liquid handling robot. It is clear that this protocol could be used to investigate small molecules that disperse biofilms, given that anti-biofilm compounds interfere with c-di-GMP signaling. The protocol could also be redeveloped to understand various aspects of bacterial physiology. Given that c-di-GMP signaling is prevalent in most bacteria, by studying the physiologies of these organisms using this protocol we can identify different chemical stimuli to determine whether or not their working mechanisms require c-di-GMP signaling.

In summary, the robustness and versatility of the approach presented in this protocol will aid the identification of chemical modulators of c-di-GMP signaling in many biological systems.

## Disclosures

The authors have nothing to disclose.
